# Impact of the *Ananya* program on reproductive, maternal, newborn and child health and nutrition in Bihar, India: early results from a quasi-experimental study

**DOI:** 10.7189/jogh.10.021002

**Published:** 2020-12

**Authors:** Gary L Darmstadt, Yingjie Weng, Kevin T Pepper, Victoria C Ward, Kala M Mehta, Evan Borkum, Jason Bentley, Hina Raheel, Anu Rangarajan, Debarshi Bhattacharya, Usha Kiran Tarigopula, Priya Nanda, Swetha Sridharan, Dana Rotz, Suzan L Carmichael, Safa Abdalla, Wolfgang Munar, for the Ananya Study Group, Yamini Atmavilas, Bill and Melinda Gates Foundation, Delhi, India, Debarshi Bhattacharya, Bill and Melinda Gates Foundation, New Delhi, India, Jason Bentley, Quantitative Sciences Unit, Department of Medicine, Stanford University School of Medicine, Stanford, CA, USA, Evan Borkum, Mathematica Policy Research, Princeton, NJ, USA, Suzan Carmichael, Department of Pediatrics, Stanford University School of Medicine, Stanford, CA, USA, Indrajit Chaudhuri, Andreea Creanga, Department of International Health, Johns Hopkins Bloomberg School of Public Health, Baltimore, MD, USA, Gary L. Darmstadt, Department of Pediatrics, Stanford University School of Medicine, Stanford, CA, USA, Priyanka Dutt, BBC Media Action, (India), New Delhi, India, Laili Irani, Population Council, New Delhi, India, Tanmay Mahapatra, CARE India, Patna, India, Kala M. Mehta, Department of Pediatrics, Stanford University School of Medicine, Stanford, CA, USA, Department of Epidemiology and Biostatistics, University of California San Francisco, San Francisco, CA, USA, Radharani Mitra, BBC Media Action, (India), New Delhi, India, Wolfgang A. Munar, Department of Global Health, George Washington University Milken Institute School of Public Health, Washington DC, USA, Priya Nanda, Bill and Melinda Gates Foundation, Delhi, India, Kevin T. Pepper, Department of Pediatrics, Stanford University School of Medicine, Stanford, CA, USA, Hina Raheel, Department of Pediatrics, Stanford University School of Medicine, Stanford, CA, USA, Anu Rangarajan, Mathematica Policy Research, Princeton, NJ, USA, Niranjan Saggurti, Population Council, New Delhi, India, Padmapriya Sastry, BBC Media Action, (India), New Delhi, India, Hemant Shah, CARE India, Patna, India, Sridhar Srikantiah, CARE India, Patna, India, Usha Kiran Tarigopula, Bill and Melinda Gates Foundation, New, Delhi, India, Victoria Ward, Department of Pediatrics, Stanford University School of Medicine, Stanford, CA, USA, Yingjie Weng, Quantitative Sciences Unit, Department of Medicine, Stanford University School of Medicine, Stanford, CA, USA, Dilys Walker, Obstetrics and Gynecology and Global Health Sciences, University of California San Francisco, San Francisco, CA, USA, Jess Wilhelm, Department of International Health, Johns Hopkins Bloomberg School of Public Health, Baltimore, MD, USA

**Affiliations:** 1 Department of Pediatrics, Stanford University School of Medicine, Stanford, California, USA; 2 Center for Population Health Sciences, Stanford University School of Medicine, Palo Alto, California, USA; 3 Quantitative Sciences Unit, Department of Medicine, Stanford University School of Medicine, Stanford, California, USA; 4 Department of Epidemiology and Biostatistics, University of California San Francisco, San Francisco, California, USA; 5 Mathematica, Princeton, New Jersey, USA; 6 Bill & Melinda Gates Foundation, Delhi, India; 7 Department of Global Health, George Washington University Milken Institute School of Public Health, Washington, D.C., USA

## Abstract

**Background:**

The Government of Bihar (GoB) in India, the Bill and Melinda Gates Foundation and several non-governmental organisations launched the *Ananya* program aimed to support the GoB to improve reproductive, maternal, newborn and child health and nutrition (RMNCHN) statewide. Here we summarise changes in indicators attained during the initial two-year pilot phase (2012-2013) of implementation in eight focus districts of approximately 28 million population, aimed to inform subsequent scale-up.

**Methods:**

The quasi-experimental impact evaluation included statewide household surveys at two time points during the pilot phase: January-April 2012 (“baseline”) including an initial cohort of beneficiaries and January-April 2014 (“midline”) with a new cohort. The two arms were: 1) eight intervention districts, and 2) a comparison arm comprised of the remaining 30 districts in Bihar where *Ananya* interventions were not implemented. We analysed changes in indicators across the RMNCHN continuum of care from baseline to midline in intervention and comparison districts using a difference-in-difference analysis.

**Results:**

Indicators in the two arms were similar at baseline. Overall, 40% of indicators (20 of 51) changed significantly from baseline to midline in the comparison districts unrelated to *Ananya*; two-thirds (n = 13) of secular indicator changes were in a direction expected to promote health. Statistically significant impact attributable to the *Ananya* program was found for 10% (five of 51) of RMNCHN indicators. Positive impacts were most prominent for mother’s behaviours in contraceptive utilisation.

**Conclusions:**

The *Ananya* program had limited impact in improving health-related outcomes during the first two-year period covered by this evaluation. The program’s theories of change and action were not powered to observe statistically significant differences in RMNCHN indicators within two years, but rather aimed to help inform program improvements and scale-up. Evaluation of large-scale programs such as *Ananya* using theory-informed, equity-sensitive (including gender), mixed-methods approaches can help elucidate causality and better explain pathways through which supply- and demand-side interventions contribute to changes in behaviour among the actors involved in the production of population-level health outcomes. Evidence from Bihar indicates that deep structural constraints in health system organisation and delivery of interventions pose substantial limitations on behaviour change among health care providers and beneficiaries.

**Study registration:**

ClinicalTrials.gov number NCT02726230.

The Sustainable Development Goals (SDGs) and recent calls for Universal Health Coverage (UHC) have reaffirmed global commitments to ensure affordable and equitable access to high-quality health services for all people. Strengthening primary health care (PHC) systems has been well-established as a necessary condition for achieving these goals [1]. Moreover, it has been estimated that investing in high-quality health systems could prevent eight million deaths each year in low- and middle-income countries (LMICs) [2].

Improving PHC system performance in LMICs remains a major global health challenge [2-5]. Poorly performing health systems, characterised by “systematic deficits in quality of care,” are common across contexts [5], and the effective coverage of many life-saving reproductive, maternal, newborn and child health and nutrtion (RMNCHN) interventions between and within LMICs remains variable [6-8]. Further, equity analyses capture stark variations in coverage levels between the richest and poorest groups in society [9-11].

Government-managed PHC systems are central to the provision of RMNCHN services at scale in many LMICs, including in India and the state of Bihar. Despite significant out of pocket private expenditures [12], high levels of poverty and the existence of multiple vulnerable populations (eg, Scheduled Castes, Scheduled Tribes, Pashmunda Muslims) make the role of government in the provision of targeted RMNCHN services critical. Moreover, marked underperformance in RMNCHN population-level indicators also makes strengthening of governmental capabilities paramount [13].

There is a robust body of evidence assessing the impacts of multiple RMNCHN interventions delivered through PHC platforms, including (a) community health workers, (b) health facilities, (c) social and behavioural change communication programs, (d) women’s self-help groups (SHGs), and (e) other community-based organisations [14-27]. While various RMNCHN interventions have been proven to be efficacious in reducing the burden of maternal and child mortality and morbidity, their scale up and sustainability remain challenging in many low-income country contexts.

Limited rigorous quantitative evidence is available on the impact of large-scale, multi-faceted RMNCHN programs in the Indian context, particularly programs that use multiple delivery channels and seek to improve outcomes across the RMNCHN continuum of care [28-34]. In one program, CARE (Cooperative for Assistance and Relief Everywhere) India worked alongside the Government of India, leading to reduced neonatal mortality with increases in postnatal frontline worker (FLW) home visits [35]. Another program in India (*Sure Start*) involved a partnership between PATH and the Government of Uttar Pradesh and generated significant improvements in care-seeking and healthy behaviours through community-level interventions, including communications, strengthening of local health committees, and mobilisation and mentoring of FLWs [36].

*Ananya* was a comprehensive, complex RMNCHN program in Bihar, India that was piloted at significant scale (28 million population across eight of 38 districts) during 2012-2013. The program was funded by the Bill & Melinda Gates Foundation (BMGF) and involved several non-governmental organisations (NGOs) working with the Government of Bihar (GoB) to strengthen PHC delivery and improve RMNCHN-related practices. While the *Ananya* program had an over-arching theory of change [13], each partner within the program had their specific theories of action and implementation plans for designing and piloting specific supply-side [eg, CARE India through the Integrated Family Health Initiative (IFHI)], demand-side [eg, BBC Media Action through the Shaping Demand and Practices (SDP) grant] and community-side (eg, Project Concern International through the *Parivartan* self-help group project) innovations. Here we describe the results from a quasi-experimental study using data which were independent of program support to examine changes in RMNCHN-related behaviours across the continuum of care and intervention delivery platforms before (baseline) and after two years of implementation of the *Ananya* program (midline).

## METHODS

### Program context and implementation

In May 2010, BMGF signed a Memorandum of Understanding with the GoB, launching the *Ananya* program to implement and test – in partnership with NGOs – a package of RMNCHN interventions in eight focus districts with the overarching goal of supporting the GoB to reduce neonatal mortality and malnutrition among women and children, as described previously [13]. The focus of the *Ananya* initiative was to accelerate progress in RMNCHN by designing and delivering a select set of evidence-based RMNCHN interventions through multiple delivery platforms that focused on the 1000-day window between the start of a mother’s pregnancy and a child’s second birthday. These interventions were designed to address and mitigate supply and demand-side constraints with the purpose of increasing availability, utilisation, quality and equity of high-impact RMNCHN interventions focused at home, community and first-level clinics.

Implementation of *Ananya* was supported by multiple grants, three of which are the focus of this paper: IFHI led by CARE India [13,37] and the SDP grant to BBC Media Action [38] for ancillary support to GoB implementation, and a grant to Mathematica to evaluate the impacts of the program. **Table 1** briefly outlines key IFHI and SDP interventions that were initially piloted in eight focus districts during 2012 and 2013, with a plan to scale up effective interventions to the other 30 comparison districts. A more comprehensive description of the IFHI and SDP interventions is provided elsewhere [13,37,38]. IFHI implemented a package of interventions at the community/outreach level and in public health facilities with the intent to: 1) improve health outcomes in the eight focus districts by increasing the number and contact time of frontline worker (FLW) [including Accredited Social Health Activist (ASHAs) and Anganwadi Worker (AWW)] home visits; 2) increase the timeliness and quality of FLW interactions with households and communities; 3) develop new tracking systems to reach marginalised groups and other beneficiaries that had historically not been visited by FLWs; 4) strengthen FLW technical capacity, knowledge, and interpersonal communication skills to confidently provide accurate and stage-specific health information; and 5) build accountability and performance management systems through strengthened supervisory structures. Although not a focus of the pilot phase, IFHI also embarked on the development of comprehensive quality improvement initiatives in public facilities to improve clinical care through structured assessments, identifying gaps in coverage, and developing action plans for systematic improvement [13,39,40]. BBC Media Action piloted a range of mass-media and social and behaviour change communication interventions designed to increase demand for and adoption of priority health behaviours at the community level [20,38]. Interventions varied in the timing and location of introduction and testing, and included mHealth-based job aids for FLWs to improve the delivery of quality health messages, as well as multimedia messaging through television, radio and street theater to encourage the adoption of healthy behaviours.

**Table 1:** Overview of *Ananya* reproductive, maternal, newborn and child health and nutrition interventions in Bihar, India, 2012-2013

Intervention	Strategic objective	Description
**Community/Outreach:**		
**Frontline worker (FLW) training (CARE India and BBC Media Action)**	Comprehensive capacity building for FLWs	• Health subcentre platform meetings: integrated delivery platform to improve last-mile service delivery by bringing all FLWs at the health sub-center level together on a fixed day to improve planning, coordination, skill development, and use of data
		• Name-based tracking: system to ensure that key health services reach all households
		• Surveillance systems: Track serious health events and facilitate referrals and care
**Mass media campaign (BBC Media Action)**	Generate increased demand for health services through comprehensive mass-media information campaigns	• Increase demand for and adoption of priority health behaviours at the community level through a “360-degree” communication strategy, which included multiple, complementary channels intended to saturate communities with priority reproductive, maternal, newborn and child health and nutrition messages. Mass media interventions included community campaigns focused on birth spacing and complementary feeding.
**Public health facilities:**		
**Quality improvement**	Implementation of quality improvement (QI) teams to incrementally drive facility-based improvements	• Infrastructure: Basic equipment (eg, delivery tables, blood pressure cuffs, weighing scales)
		• Supplies: Medications and sanitation tools (eg, gloves, basic drugs)
		• Documentation and record keeping
		• Implementation of clinical care practices: infection control, basic care of newborns and mothers (ie, vital signs, skin-to-skin care, exclusive breastfeeding, warning sign identification)
		• Management: Utilisation of dashboards, checklists, complication review, and referral tracking
**Nurse mentoring**	Nurse mentoring and training for facility staff (started in mid-2013)	• Skills laboratories, mentoring and on-the-job training for management of complications, stabilisation referrals, documentation and tracking

*Ananya* was originally designed as a pilot program to inform the subsequent GoB-led statewide scale-up (from eight to all 38 districts in Bihar) of prioritised RMNCHN interventions and platforms. After two years of implementation, the GoB prompted an accelerated scale-up, and in late 2013, CARE India formed the Bihar Technical Support Program (BTSP) to partner with the GoB to strengthen the Bihar public health system. A description of the BTSP – including goals, interventions, grantees, and data sources – m is provided elsewhere [13]. Thus, the GoB-BMGF-NGO partnership consisted of two phases: (1) two full years of intensive program implementation support to the government (2012-2013) in eight focus districts, termed *Ananya* – the pilot phase – followed by (2) transition to less intensive techno-managerial support to the GoB for statewide scale-up across 38 districts and 104 million population (2014 to present), complemented by other initiatives as described previously [13].

### Evaluation design

Mathematica implemented a quasi-experimental impact evaluation of *Ananya* that included statewide household surveys at two time points: January through April 2012 (“baseline”) and January through April 2014 (“midline”) [41]. An original plan for an “endline” survey approximately five years into the program became unfeasible when the GoB began to scale-up select interventions statewide in all districts in 2014. The two arms of the evaluation included the intervention arm comprised of the eight focus districts, and a comparison arm that included the remaining 30 districts in Bihar where *Ananya* was not being implemented during the pilot phase. The eight focus districts in the pilot phase were located in a cluster in the northwest region of the state (East Champaran, West Champaran, and Gopalganj) and in another that was relatively accessible near the capital city of Patna (Patna, Samastipur, Begusarai, Saharsa, and Khagaria) [13].

### Sampling design and survey procedures

Mathematica surveyors collected data from households using a three-stage sampling design applied at baseline, and returned to the same villages to collect surveys at midline, although from a different cohort of women. In the first stage, a representative sample of blocks (the primary sampling unit, or PSU) was randomly selected in each district with larger districts including proportionally more PSUs. Stratification sampling by urban/rural area was performed to enrich the urban population in the sample. In the second stage, a representative set of secondary sampling units (SSUs) in the sampled PSUs was identified, with proportionally more SSUs identified in larger PSUs. SSUs were also defined as villages in rural areas and blocks in urban areas. Small SSUs (those with fewer than 75 households) were combined with nearby SSUs into a single SSU before sampling. In the third stage, large rural SSUs (those with 150 households or more) were first divided into several equal-sized segments of 75 to 150 households per segment. A single segment was then randomly selected into the sample; urban SSUs were rarely much larger than 100 households, and thus, this step was necessary only for rural SSUs.

Surveys were administered to maternal household respondents who had given birth in the catchment areas in the previous year. Surveys were conducted by an independent contractor (Sambodhi) in collaboration with Mathematica. Surveys focused on children ages 0-11 months because interventions were targeted most intensively on delivery and postnatal infant outcomes in the first year after delivery. Mathematica did not conduct longitudinal follow-up of the same cohort of women at baseline and midline because outcomes were focused on behaviours and practices at a particular stage of life, and women from the baseline cohort may or may not have had another child in the year prior to the midline survey; rather cross-sectional surveys were administered and thus the maternal respondents at baseline were not the same cohort as for the midline but were sampled from the same villages and segments. Mathematica considered various options and selected all non-focus districts (n = 30), where the Ananya program had not been implemented, as the primary comparison group. Comparing focus and comparison districts across both the 2012 and 2014 surveys enabled estimation of difference-in-difference as a reflection of the contribution of the *Ananya* program to changes in indicators over the survey period, assuming that trends in treatment and comparison would have been the same in the absence of the program, as suggested by prior Mathematica analysis [41]. This, then, also enabled assessment of changes from baseline to midline in the absence of *Ananya* interventions. To account for the most appropriate survey design scheme, the analysis specified the district as the first level of sampling, block as the second level (with urban/rural categorisation as stratum and appropriate finite population corrections within each stratum), and villages or urban blocks as the third level. Household-level sampling weights were also applied, which accounted for all the stages of sampling.

### Data analysis

#### RMNCHN indicator selection and categorisation

RMNCHN indicators (n = 51) that directly reflected the multi-faceted *Ananya* program were pre-specified for analysis based on review by three independent members of the Stanford analytic team with expertise in maternal and child health and the conduct of field research (Table S1 in the **Online Supplementary Document**). While we attempted to use all indicators measured (a census), some indicators were ultimately not used because: 1) they were redundant (more than one variable was assessed for the same indicator), 2) they were judged unfit due to lack of specificity of the question, 3) the item was judged unfit due to poor quality of data obtained, and 4) harmonisation was not possible due to changes in the question stem across surveys.

Indicators were first grouped into the following domains according to the continuum of care: antenatal care (ANC), birth preparedness, delivery (childbirth care), postnatal care, child nutrition/complementary feeding, child immunisation, and family planning (Table S2 in the **Online Supplementary Document**). Within each of these domains, we further classified the indicators into three delivery platforms or approaches: FLW performance or behaviour, mother’s behaviour, and facility care and outreach service delivery (recognising the limited emphasis on facility-based care in the pilot phase). Our aim was to characterise program impact based on continuum of care domains and delivery platforms by examining trends for subgroups of indicators. Indicators of FLW performance were based on actions carried out by FLWs, for example giving advice on various aspects of pregnancy and newborn care and conducting postnatal visits. Indicators of mother’s behaviour were heavily dependent on her decision to adopt that behaviour, with a less tangible role for the FLW or a response from the health system. Indicators of facility care and outreach service delivery were those that reflected the quality of supply chains and availability and quality of facility-based care such as ANC, hygienic practice of birth attendants in facility-based deliveries, provision of iron-folic acid (IFA) tablets, family planning procedures and immunisations.

#### Statistical analysis

We examined the demographic characteristics of maternal respondents by their treatment allocation (focus/intervention vs non-focus/comparison district) and survey time (baseline vs midline). We reported crude percentages without adjusting for survey design or weights.

For each of the RMNCHN indicators, two multivariate regression models that accounted for the survey design were first constructed to compare the difference between the intervention and comparison by baseline and midline, respectively. To determine the effect attributable to the *Ananya* program, we conducted difference-in-difference (DID) analysis to model the intervention effect, accounting for the survey design [42]. The independent variables were the binary intervention group (focus vs comparison districts), study period (baseline vs midline), and an interaction term of these two factors. The DID estimator from the model is the interaction term that captured the change in reported RMNCHN indicators among women respondents attributed to the *Ananya* program. All three models (baseline, midline and DID model) were adjusted for potential confounding variables, including maternal age, maternal respondents’ religion (Hindu vs non-Hindu), whether a woman belonged to a Scheduled Tribe or Scheduled Caste (STSC), number of children, household size, literacy, and socioeconomic status (SES) quartile. SES quartile was determined using methods based on the National Family Health Survey (NFHS)-3 [43]. Principal components analysis on the baseline data was used to compute a wealth index for each household based on characteristics likely to reflect poverty, such as the number of household members per room, the material from which the residence was constructed, and ownership of various durable goods. Coefficients from the baseline principal components analysis were used to estimate the wealth index for each woman at midline. The first principal component explained 16.5% of the variability (data not shown). Quartiles are relative to the 2012 statewide SES distribution for women who gave birth in the previous 12 months. We used survey Poisson regressions for count-type indicators while survey logistic regressions were used for binary indicators. We further evaluated and reported the percentage point difference of the DID estimators by estimating the marginal effect of the interaction term from the logistic regression models. Forest plots presenting DID estimators and their 95% confidence intervals (CIs) were used to summarise the impact of the *Ananya* program by key indicator domains (ie, across the continuum of care and delivery platforms). We also conducted exploratory analyses using the same analytic approach to test the hypothesis that sub-groups of women who delivered in facilities compared to homes, or had received either two or more third-trimester antenatal FLW home visits or one or more early postnatal FLW home visits would show differences in selected indicators compared to women who did not receive these aspects of care. For these exploratory analysis, *P* values for the regression models were reported and FDR adjustment was not done since the analysis was post-hoc. Associations between intervention group and RMNCHN indicators were assessed at alpha = 0.05. Analyses were conducted in Stata version 14 [44]. Forest plots were produced via ‘ggplot2’ package in R 3.4.3 [45,46]. Due to the large number of comparisons, we applied the False Discovery Rate (FDR) controlling procedure by Benjamini and Hochberg [47] using SAS proc multtest, which reduces the false positive (type I error) rate by applying an upward adjustment to the *P*-values.

### Ethical considerations

This study is part of the *Ananya* Bihar program and is registered at ClinicalTrials.gov number NCT02726230. The Stanford Institutional Review Board gave ethical approval for the analyses through protocol 39719.

## RESULTS

### Study population demographic characteristics

Characteristics of survey respondents were similar in focus and comparison districts at baseline and at midline (**Table 2****)**. The average maternal respondent was approximately 26 years old, four-fifths (82%) were Hindu, about one-quarter belonged to Scheduled Caste/Scheduled Tribe, about 60% at baseline and 50% at midline had no formal education and about 40% at baseline and 45% at midline were literate. The median household size was about six, 52%-54% of the focal children of the maternal respondents were male, and about one-third of husbands had more than one year of formal education.

**Table 2:** Characteristics of survey respondents with infants 0-11 months old in the *Ananya* program focus (intervention) and comparison districts at baseline and at midline, Bihar, India*

	Baseline, January – April, 2012		Midline, January – April, 2014	
**Characteristic** (%)†	**Baseline**		**Midline**	
**Maternal characteristics**	**Intervention**	**Comparison**	**Intervention**	**Comparison**
Age (years), mean (standard deviation)	25.8 (4.4)	25.9 (4.9)	25.5 (4.4)	25.5 (4.4)
Hindu	82	81	82	83
Scheduled caste/Scheduled tribe (subset of Hindu)	23	26	32	24
No formal education	61	59	51	50
Literate (can read and write)	39	38	45	47
Birth parity:				
1 child	29	32	20	31
2 children	26	28	27	27
3 children	22	19	21	21
4 or more children	23	22	24	22
**Household characteristics**				
Household size (number), median (interquartile range)‡	6 (4-7)	6 (4-7)	6 (4-7)	5 (4-7)
Nuclear family type	51	50	52	59
Gender of focal child:				
-Male	52.4	52.0	53.9	53.7
-Female	47.6	48.0	46.1	46.3
Husband ever attended school (≥1 y of education)	65	65	69	70
**Socioeconomic status (SES) quartile§**				
Quartile 1	23	26	29	29
Quartile 2	24	25	18	21
Quartile 3	27	25	25	24
Quartile 4	27	25	28	27

*Mathematica data, 2012 and 2014.

†Percentage unless otherwise specified.

‡Median used due to non-normal data.

§SES quartile determined using National Family Health Survey-3 methods.

### Indicators at baseline

**Table 3** shows baseline and midline results for focus and comparison districts and Table S3 in the **Online Supplementary Document** shows corresponding sample sizes. Indicators in focus and comparison districts were similar at baseline (**Table 3**), although some minor differences were present. At baseline, home births were higher in the comparison (40%) than the intervention districts (32%) whereas public facility births were lower in the comparison (45%) than the intervention districts (53%). Delayed newborn bathing was lower in the comparison (46%) than intervention districts (55%) but exclusive breastfeeding was higher in comparison districts (44% vs 39%) and wasting at 9-11 months of age was higher in comparison districts (40% vs 35%). Some family planning indicators were slightly higher in comparison than intervention districts. Overall, differences attributable to the program were driven by differences at midline, after two years of program implementation, and furthermore, DID analysis took baseline differences into account.

**Table 3:** Differences attributable to the *Ananya* program for maternal household respondents with infants 0-11 months old on selected reproductive, maternal, newborn, and child health and nutrition indicators in Bihar, India, Mathematica data, 2012 and 2014

	Baseline, 2012		Midline, 2014		Secular Change*		Difference-in-Difference attributable to *Ananya* interventions	
	**Comparison** (n = 9406)	**Intervention** (n = 2978)	**Comparison** (n = 8562)	**Intervention** (n = 3092)	**Effect size, percentage point difference (95% confidence interval)**	***P*-value**	**Difference-in-dsifference, percentage point difference (95% confidence interval)**	***P*-value†**
**ANTENATAL CARE (%)**								
**Antenatal checkups:**								
>4 antenatal care checkups	13	16	18	24	3.8 (1.4, 6.3)	0.003	2.8 (-9.0, 6.5)	0.35
1+ blood pressure measurements during pregnancy	53	51	50	55	-3.6 (-8.0, 0.8)	0.108	6.7 (-0.2, 13.5)	0.26
>2 frontline worker (FLW) visits in the last trimester	36	33	32	39	-3.2 (-6.9, 0.3)	0.074	9.8 (3.2, 16.4)	0.041
Iron-folic acid tablets:								
Received 90 + tablets	18	17	17	17	-1.0 (-4.3, 2.3)	0.558	0.9 (-5.6, 7.4)	0.87
Consumed 90 + tablets	12	13	15	15	2.6 (0.2, 5.1)	0.033	0.0 (-6.9, 7.0)	0.98
**BIRTH PREPAREDNESS (%)**								
Identified place of delivery	68	65	39	38	-26.7 (-32.9, -20.7)	<0.001	2.3 (-7.4, 12.1)	0.83
Identified skilled birth attendant (home deliveries)	60	64	51	46	-9.0 (-18.3, 0.3)	0.059	-8.5 (-26.5, 9.6)	0.67
Saved money for delivery	75	75	75	79	0.5 (-3.9, 5.0)	0.808	4.7 (-1.2, 10.6)	0.38
Identified transportation to facility	59	60	55	60	-3.3 (-9.5, 2.9)	0.287	4.2 (-3.9, 12.2)	0.63
Pregnancy registration	75	72	84	81	9.5 (4.8, 14.2)	<0.001	-1.0 (-8.0, 5.9)	0.87
**DELIVERY (%)**								
Received *Janani Avam Bal Suraksha Yojana* (JBSY) payment	47	44	66	68	19.8 (14.4, 25.2)	<0.001	3.5 (-7.3, 14.3)	0.75
**Place of delivery:**								
Home delivery	40	32	29	22	-10.1 (-13.7, -6.6)	<0.001	-0.7 (-5.0, 3.6)	0.87
Facility delivery: Public	45	53	55	61	10.3 (6.9, 13.6)	<0.001	-2.4 (-8.2, 3.3)	0.69
Facility delivery: Private	16	15	16	17	-0.5 (-1.8, 0.9)	0.477	2.1 (-0.3, 4.7)	0.27
**POSTNATAL CARE (%)**								
**Postnatal FLW home visits:**								
Postnatal visit within 48 hours	11	12	13	15	2.2 (-0.2, 4.6)	0.071	-0.3 (-4.8, 4.2)	0.96
Postnatal visit within 7 days	17	19	17	19	-0.5 (-3.3, 2.4)	0.737	-0.1 (-5.6, 5.3)	0.98
**Postnatal FLW counseling:**								
Skin-to-skin (kangaroo) care	33	36	29	38	-3.8 (-9.6, 1.9)	0.180	8.0 (-6.8, 22.7)	0.57
Exclusive breastfeeding	39	42	32	43	-6.1 (-11.5, -0.6)	0.029	7.9 (-3.5, 19.4)	0.41
**Neonatal health-related behaviours:**								
Baby was immediately wiped dry and wrapped	69	72	86	87	16.4 (12.4, 20.5)	<0.001	0.5 (-7.8, 8.8)	0.96
Nothing applied to cord or umbilicus	24	24	23	30	-1.4 (-4.8, 2.0)	0.413	7.5 (1.5, 13.4)	0.07
Skin-to-skin contact‡	18	20	32	43	13.8 (4.7, 22.9)	0.005	8.1 (-12.8, 2.9)	0.70
First bath delayed by 2 days	46	55	54	65	7.8 (3.4, 12.1)	0.001	2.5 (-6.3, 11.2)	0.72
Breastfed within 1 h of birth	44	47	46	52	2.0 (-0.5, 4.4)	0.112	2.9 (-5.0, 10.8)	0.71
Exclusive breastfeeding for 6 months	44	39	48	53	4.3 (-1.6, 10.2)	0.150	10.0 (-0.6, 20.7)	0.26
**CHILD NUTRITION/COMPLEMENTARY FEEDING (%):**								
**Complementary feeding (among children aged 6-11 months):**								
Currently receiving any solid or semisolid food§	65	65	61	68	-4.7 (-8.6, -0.8)	0.020	7.9 (-1.3, 17.1)	0.34
Began receiving any solid or semisolid food by age 6 months	49	48	39	45	-9.7 (-13.8, -5.6)	<0.001	7.3 (0.1, 14.6)	0.26
**Undernutrition in children:**								
Stunted (low height for age)‖	32	33	34	29	1.5 (-1.0, 4.0)	0.226	-5.9 (-10.2, -1.7)	0.066
Wasted (low weight for height)¶	40	35	38	31	-3.4 (-7.4, 0.6)	0.09	-1.1 (-8.3, 6.1)	0.87
Underweight (low weight for age)**	31	27	27	24	-4.4 (-7.8, -1.0)	0.016	0.9 (-6.5, 8.3)	0.89
**CHILD IMMUNISATION (%)**								
Immunisation card available	45	44	52	53	6.1 (1.9, 10.4)	0.006	2.4 (-4.7. 9.6)	0.74
No immunisations given (unvaccinated)	8	9	4	6	-4.1 (-5.8, -2.3)	<0.001	1.4 (-1.9. 4.7)	0.68
Individual immunisations††								
Bacillus Calmette–Guérin (BCG)	87	85	91	88	3.5 (1.3, 5.7)	0.004	-1.6 (-5.8, 2.7)	0.71
Diphtheria-pertussis-tetanus (DPT)3	38	35	36	35	-2.7 (-5.7, 0.3)	0.079	3.0 (-5.0, 10.9)	0.71
Measles	11	13	9	11	-2.2 (-3.8, -0.5)	0.010	1.0 (-4.7, 6.7)	0.87
Polio3	38	36	37	36	-1.1 (-3.8, 1.6)	0.405	2.4 (-5.7, 10.5)	0.75
Fully vaccinated (but no measles)	32	30	32	29	-0.5 (-3.2, 2.3)	0.717	0.3 (-8.7, 9.2)	0.98
Fully vaccinated	10	12	7	9	-2.8 (-4.4, -1.0)	0.002	0.9 (-4.9, 6.8)	0.87
**FAMILY PLANNING (%)**								
Plans to use any modern method of contraception in the next 12 months‡‡	48	49	46	42	-1.6 (-6.9, 3.7)	0.543	-6.3 (-16.8, 4.2)	0.54
**Procedures performed after delivery:**								
Postpartum sterilisation/tubal ligation	3.7	1.8	3.0	3.0	-0.4 (-1.7, 0.9)	0.540	3.0 (0.6, 5.4)	0.026
Postpartum intrauterine device (IUD)/Copper-T	2.6	3.9	0.4	0.2	-2.2 (-2.9, -1.5)	<0.001	-1.3 (-2.3, -0.3)	0.26
**Use of any modern method of contraception (by age of child):**								
0-5 months	12	10	12	20	0.7 (-2.2, 3.6)	0.616	11 (2.6, 18.8)	0.034
6-11 months	17	13	16	19	-0.1 (-3.2, 2.9)	0.928	7.9 (-2.0, 17.8)	0.27
**Use of any modern method of contraception (by method):**								
Condom	3.3	2.5	3.4	6.0	0.2 (-1.0, 1.4)	0.769	3.9 (-3.2, 10.9)	0.41
Intrauterine device	1.6	2.0	0.6	0.4	-0.8 (-1.8, 0.1)	0.055	-0.5 (-1.9, 0.9)	0.76
Pills	2.7	2.5	1.6	5.1	-1.0 (-2.1, 0.2)	0.098	5.3 (-0.6, 11.3)	0.06
Tubal ligation	7.5	5.6	8.1	7.4	1.1 (-0.4. 2.7)	0.152	1.7 (-1.3, 4.7)	0.50
Injectables	0.3	0.3	0.2	0.6	-0.04 (-0.3, 0.3)	0.797	0.3 (-0.7, 1.4)	0.70
**Use of any modern method of contraception (by parity):**								
Mothers with 1 child	8	8	7	14	-1.0 (-4.8, 2.6)	0.577	8.2 (-1.7, 18.0)	0.26
Mothers with >1 child	17	12	17	22	0.7 (-1.6, 2.9)	0.541	10 (3.0, 17.8)	0.038
**Use of any modern method of contraception (by gender of most recent child):**								
Male	15	14	16	19	1.7 (-1.3, 4.7)	0.245	4.6 (-2.7, 12.0)	0.44
Female	13	8.0	12	20	-0.3 (-2.7, 2.1)	0.791	18 (6.9, 28.2)	<0.001

*Change from baseline to midline in the comparison group.

†FDR-adjusted *P*-value.

‡When the baby is placed unclothed on mother’s chest or abdomen with skin-to-skin contact under a blanket or some clothing

§Cereal-based food (rice, khichdi, or bread).

‖Stunting–height-for-age z-score below 2 standard deviations (SDs) from the median height for age of the international reference population (which was drawn from six diverse countries).

¶Wasting: weight-for-height z-score below 2 SDs of the reference population median.

**Underweight: weight-for-age z-score below 2 SDs of the reference population median.

††Immunisation based on card and self-report, combined.

‡‡Only for women who are not currently using any modern method of contraception.

### Changes in indicators in comparison districts without *Ananya* interventions

Examination of changes in indicators in comparison districts from baseline to midline enabled insights into secular changes in indicators across the continuum of care in the absence of direct implementation of *Ananya* interventions (**Table 3****)**. Significant health-promoting changes from baseline to midline in comparison districts were observed for 13 indicators, including four or more ANC checkups [four percentage points (ppt) increase, *P* = 0.003), pregnancy registration (ten ppt increase, *P* < 0.001), consumption of 90 or more IFA tablets during pregnancy (three ppt increase, *P* = 0.033), home deliveries (ten ppt decrease, *P* < 0.001), public facility deliveries (ten ppt increase, *P* < 0.001), receipt of conditional cash transfer payment through *Janani Avam Bal Suraksha Yojana* (JBSY) for facility delivery (20 ppt increase, *P* < 0.001), immediate wiping and drying of the newborn (16 ppt increase, *P* < 0.001), skin-to-skin contact (14 ppt increase, *P* = 0.005), delay of first bath by ≥2 days (eight ppt increase, *P* = 0.001), underweight (four ppt decrease, *P* = 0.016), immunisation card available (six ppt increase, *P* = 0.006), unvaccinated infants (four ppt decrease, *P* < 0.001), and Bacillus Calmette–Guérin (BCG) immunisation (four ppt increase, *P* = 0.004). In contrast, there were significant reductions in seven indicators of health promotion from baseline to midline, including identification of the place of delivery as part of birth preparedness (27 ppt decrease, *P* < 0.001), exclusive breastfeeding (six ppt decrease, *P* = 0.029), complementary feeding [ie, currently receiving any solid or semisolid food (five ppt decrease, *P* = 0.02), began receiving any solid or semisolid food by age 6 months (ten ppt decrease, *P* < 0.001)], measles immunisation (two ppt decrease, *P* = 0.01), fully vaccinated (three ppt decrease, *P* = 0.002), and use of a postpartum intrauterine device for contraception (two ppt decrease, *P* < 0.001). Thus, changes in indicators associated with contextual factors unrelated to *Ananya* were mixed, but overall favoured improvements in indicators in the direction of health promotion. These changes were also accounted for in DID analyses (ie, final column, **Table 3**).

### Program effects by continuum of care domains

**Figure 1:**
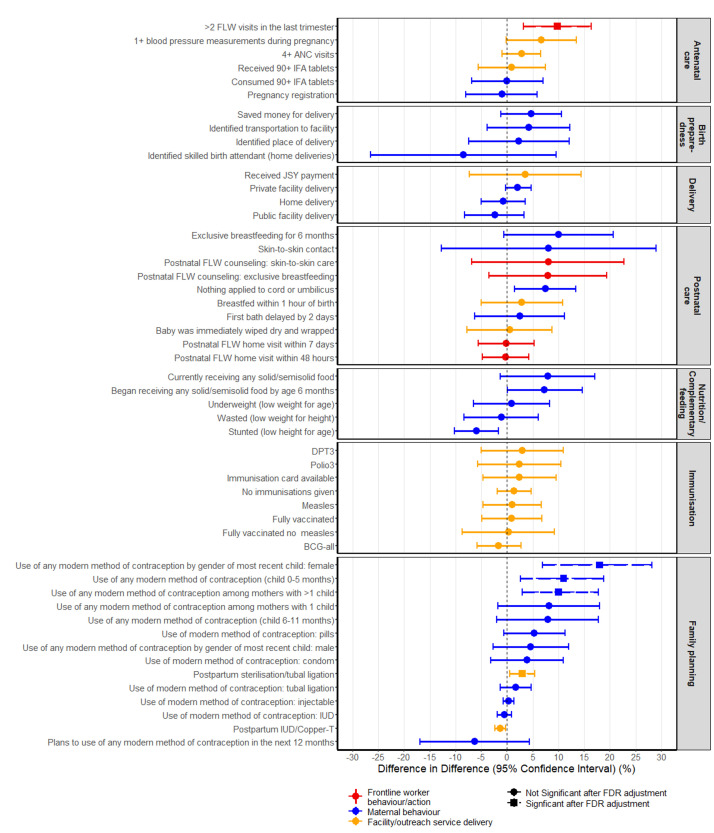
Forest plot for difference-in-difference estimators for reproductive, maternal, newborn and child health and nutrition indicators across the continuum of care for *Ananya* program focus (intervention) and comparison districts at baseline (2012) and at midline (2014), Bihar, India, Mathematica data from maternal respondents with infants 0-11 months old. ANC – antenatal care, BCG – Bacillus Calmette-Guérin, DPT – diphtheria-pertussis-tetanus, FLW – frontline worker, IFA – iron-folic acid, IUD – intrauterine device, JSY – *Janani Avam Bal Suraksha Yojana.*

Some improvements in DID estimators were seen for ANC and birth preparedness, postnatal care, child immunisation and family planning (**Figure 1**), but only five indicators (10%) showed evidence of a statistically significant improvement attributable to the *Ananya* program beween 2012 and 2014 (**Table 3**). One of ten ANC and birth preparedness indicators showed a statistically significant increase attributable to the program after FDR adjustment of *P* values: a ten ppt increase was observed in two or more FLW home visits in the last trimester *(P =* 0.041). No significant change attributable to *Ananya* was seen for four delivery indicators, ten postnatal care indicators, five variables reflecting child nutrition, and eight child immunisation indicators. Four of 14 family planning indicators improved significantly as a result of *Ananya* interventions. There was a significant three ppt increase in postpartum tubal ligation (*P* = 0.026) and significant increases in use of any modern method of contraception among mothers of younger children 0-5 months (11 ppt, *P* = 0.034) and for mothers who had more than one child (ten ppt, *P* = 0.038), with the largest increase in use of oral contraceptive pills (five ppt) although this change was borderline in significance *(P =* 0.06). The increase in contraception was significant among mothers of female children (18 ppt increase, *P* < 0.001) but not among mothers of males.

### Program effects on facility and home births

**Table 4** shows changes in newborn health behaviors between intervention and comparison districts by place of delivery (home, public facility or private facility). Among public facility births there were significant eight ppt increases in the intended practices of applying nothing to the cut umbilical cord *(P =* 0.017) and immediate breastfeeding (within one hour of birth) *(P =* 0.032) attributable to the *Ananya* program. Among private facility births, there was a 27 ppt increase in applying nothing to the cut umbilical cord *(P <* .001). No significant changes in practices attributable to *Ananya* were seen for home births.

**Table 4:** Differences attributable to the *Ananya* program on postnatal care indicators by place of delivery (home or public facility or private facility) for maternal household respondents with infants 0-11 months old in Bihar, India*

	Baseline 2012		Midline 2014		Difference-in-difference attributable to *Ananya* interventions	
	**Comparison**	**Intervention**	**Comparison**	**Intervention**	**Percentage point difference (95% confidence interval)**	***P*-value**
**Public facility delivery, N**	4186	1479	5453	2199		
Baby was immediately wiped dry and wrapped (%)	79	79	90	92	-2.3 (-4.3, 8.9)	0.508
Nothing applied to cord or umbilicus (%)	22	22	23	32	8.1 (1.1, 15)	0.017
Skin-to-skin contact (%)	20	17	36	46	14 (-6.8, 35)	0.163
First bath delayed by 2 d (%)	51	59	59	69	2.8 (-5.9, 11)	0.522
Breastfed within 1 h of birth (%)	51	52	51	59	7.8 (0.8, 15)	0.032
**Private facility delivery, N**	1626	477	1586	557		
Baby was immediately wiped dry and wrapped (%)	72	66	89	85	-2.2 (-12.6, 8.2)	0.658
Nothing applied to cord or umbilicus (%)	21	13	14	26	27 (17, 37)	<0.001
Skin-to-skin contact (%)	24	34	33	45	0.5 (-25, 25)	0.969
First bath delayed by 2 d (%)	54	55	63	64	0.2 (-12, 13)	0.971
Breastfed within 1 hour of birth (%)	44	45	34	36	1.6 (-16, 19)	0.857
**Home delivery, N**	3594	1022	2417	693		
Baby was immediately wiped dry and wrapped (%)	58	62	76	77	-3.0 (-14.7, 8.8)	0.607
Nothing applied to cord or umbilicus (%)	28	31	28	30	-2.1 (-11.5, 7.4)	0.662
Skin-to-skin contact (%)	14	16	24	32	4.2 (-15, 24)	0.644
First bath delayed by 2 d (%)	37	47	41	55	2.9 (-8.9, 15)	0.616
Breastfed within 1 h of birth (%)	36	40	44	44	-4.1 (-17, 8.4)	0.514

*Mathematica data, 2012 and 2014.

### Program effects by reach

We further examined differences in indicators attributable to *Ananya* program interventions by restricting our analyses to maternal respondents who received either two or more antenatal FLW home visits in the last trimester (**Table 5**) or one or more FLW home visits within seven days of delivery (**Table 6**). In general, there was little evidence for improved practices among women reached through antenatal (**Table 5**) or postnatal (**Table 6**) FLW home visits compared to the entire sample of maternal respondents (**Table 3**). Among women who received two or more antenatal FLW home visits, there was a significant 17 ppt *(P =* 0.038) improvement in exclusive breastfeeding but a 15 ppt *(P =* 0.04) decrease in identification of a skilled attendant for delivery among home births (**Table 5**). Among women who received a FLW home visit within seven days of delivery, there was a significant 30 ppt *(P =* 0.006) increase in exclusive breastfeeding but an 11 ppt *(P =* 0.046) decrease in immediate wiping and drying of the newborn after delivery (ie, a decrease in intended practice) (**Table 6**).

**Table 5:** Differences attributable to the *Ananya* program on antenatal, delivery and early postnatal indicators for maternal household respondents with infants 0-11 months old who received 2 or more antenatal frontline worker home visits in the last trimester in Bihar, India*

	Baseline, 2012			Midline, 2014			Difference-in-difference attributable to *Ananya* interventions		
	**Comparison** (3229)	**Intervention** (926)	**Comparison** (2633)		**Intervention** (1127)	**Percentage point difference (95% confidence interval)**		***P*-value**
**BIRTH PREPAREDNESS (%)**								
Identified place of delivery	71	70	50		57	9.2 (-2.1, 20.6)		0.129
Identified skilled birth attendant (for home delivery)	66	75	65		56	-15.1 (-29.9, -0.2)		0.040
Saved money for delivery	77	80	78		86	5.6 (-1.3, 12.4)		0.130
Identified transportation to facility	60	60	58		64	5.9 (-5.8. 17.6)		0.322
Pregnancy registered	94	92	94		95	2.1 (-1.8, 6.0)		0.324
**DELIVERY (%):**								
Received *Janani Avam Bal Suraksha Yojana* (JBSY) payment	57	49	75		77	8.1 (-3.5, 19.8)		0.183
**Place of delivery:**								
Home delivery	34	29	24		19	-3.5 (-9.2, 2.2)		0.230
Facility delivery: Public	55	62	65		71	1.4 (-5.3, 8.0)		0.679
Facility delivery: Private	10	9	11		10	2.1 (-3.1, 7.4)		0.403
**POSTNATAL CARE (%)**								
Neonatal health-related behaviors:								
Baby was immediately wiped dry and wrapped	77	81	88		88	-4.1 (-16.2, 8.0)		0.474
Nothing applied to cord or umbilicus	15	14	24		28	3.4 (-5.7, 12.5)		0.439
Skin-to-skin contact*	19	20	35		51	14.0 (-8.9, 36.0)		0.200
1^st^ bath delayed by 2 days	49	56	55		66	4.9 (-7.5, 17.4)		0.432
**Breastfeeding:**								
Breastfed within 1 hours of birth	47	51	51		56	1.3 (-7.5, 10.2)		0.761
Exclusive breastfeeding for 6 months excluding water (self-report for child 6-11 months)	47	42	46		58	16.6 (1.6, 31.7)		0.038

*Mathematica data, 2012 and 2014.

**Table 6:** Differences in postnatal newborn care attributable to the *Ananya* program for maternal household respondents with infants 0-11 months old who had a postnatal home visit by a frontline worker (ASHA/AWW/ANM) within 7 days after delivery in Bihar, India*

	Baseline, 2012		Midline, 2014		Difference-in-difference attributable to *Ananya* interventions	
**Neonatal health-related behaviours (%):**	**Comparison (n = 1713)**	**Intervention (n = 534)**	**Comparison (n = 1426)**	**Intervention (n = 657)**	**Percentage point difference (95% confidence interval)**	***P*-value**
Baby was immediately wiped dry and wrapped	71	78	90	88	-11 (-22, 0.6)	0.046
Nothing applied to cord or umbilicus	15	16	17	30	9.6 (-2.2, 21.4)	0.077
Skin-to-skin contact†	22	23	36	54	17 (-6.5, 40)	0.138
1^st^ bath delayed by 2 days	35	43	54	66	4.2 (-8.0, 16.5)	0.483
Breastfeeding (%):						
Breastfed within 1 hour of birth	50	57	45	54	2.5 (-8.8, 13.9)	0.655
Exclusive breastfeeding for 6 months excluding water (self-report for child 6-11 months)†	51	35	40	57	30 (12, 47)	0.006

*Mathematica data, 2012 and 2014.

**†**Total number = 1729. At baseline, there 700 women in comparison districts and 190 women in intervention districts. At midline, there were 572 women in comparison districts and 267 women in intervention districts.

### Program effects by intervention implementation platform

Program effects were similar across the delivery platforms (Figure S1 in the **Online Supplementary Document**). Overall, one of five indicators of FLW performance (two or more FLW antenatal home visits), three family planning indicators among 30 indicators of mother’s behaviour, and one of 16 indicators of facility/outreach service delivery (postpartum tubal ligation) showed significant improvements attributable to *Ananya* after FDR adjustment. Examination of indicators categorised by both continuum of care and delivery platform revealed that the most consistent, substantial gains attributable to *Ananya* were seen in mothers’ family planning behaviours, especially in utilisation of modern contraception (Figure S2 in the **Online Supplementary Document**).

## DISCUSSION

Few significant improvements in RMNCHN indicators could be attributed to the *Ananya* program in the first two years; only 10% (five of 51) of DID estimators were significant after FDR adjustment for multiple comparisons. Improvement was greatest for indicators of family planning, which showed significant increases in 29% (four of 14) of indicators and accounted for 80% (four of five) of all indicators showing significant improvements. This might reflect relatively uniform, low levels of most family planning indicators at the beginning of the program. Only one statistically significant improvement was seen for antenatal and birth preparedness (FLW home visits), and none for delivery care, postnatal care, child nutrition or immunisation. Three of the five indicators showing improvement reflected mother’s behaviours in utilising modern contraception. Among sub-samples of infants by place of birth, improvements were found for cord care in public and private facility births but not for home births, and immediate breastfeeding improved in public but not private facilities. Overall, no improvements attributable to the *Ananya* program were seen in postnatal care practices among home births. There was limited evidence – only for exclusive breastfeeding – that antenatal or postnatal FLW home visits were associated with improvements in delivery care or newborn care, although this result must be viewed with caution given the possibility for selection bias in that characteristics of women who received these visits could have differed across the *Ananya* program and the comparison districts.

In other analyses, we found that social and behavioural change communication interventions led by BBC Media Action showed more robust and consistent improvements in desired behaviours among mothers who were exposed to mHealth interventions [38]; however, staged implementation and low exposure levels to these interventions at this stage of program implementation and scale-up likely contributed to the lack of population-level impact seen here. Moreover, while we found strong evidence for health impact associated with SHG membership, SHG interventions had not yet been scaled up by the time of the Mathematica midline evaluation [48,49].

In Bihar (and across India), there were substantial secular declines, based on population-level survey data (eg, Annual Health Survey, Sample Registration System), in maternal mortality ratio and smaller but steady reductions in infant mortality rate, neonatal morality rate, under-5 mortality rate and total fertility rate (Table S4 in the **Online Supplementary Document**). Similar to the changes in indicators in comparison districts from baseline to midline in our study, changes in health indicators in large-scale survey data were mixed but mostly showed improvements over a similar time frame. According to Annual Health Survey (AHS) data, institutional births were steadily increasing and there were small increases in measures of ANC (3+ ANC visits and consumption of IFA), immediate and exclusive breastfeeding, child immunisation (eg, children 12-23 months fully immunised) and use of modern contraception (Table S5 in the **Online Supplementary Document**). Other AHS indicators, however, such as early postnatal visits and complementary feeding practices (eg, children receiving solid or semi-solid food and breastmilk) showed little secular change during the study period. Statewide secular increases in key health measures may be attributable to multiple statewide government programs, campaigns, and political commitments to improve economic conditions and health services that were operational during this time period. For example, funding levels were increasing from the central government to the National Rural Health Mission with a focus on reaching marginalised communities with priority health interventions and increasing numbers of health workers; and the *Janani Avam Bal Suraksha Yojana* (JBSY) program provided a cash transfer to women to incentivise facility deliveries [50]. These data illustrate the importance of taking into account changes in indicators due to influences outside the program when attempting to assess impact attributable to program implementation. Without doing so in the *Ananya* program area could result overall in an over-estimation of program impact. Thus, given the varying magnitude and pace of improvement of health measures in Bihar during this period, the DID design of this evaluation was critical for assessing the attributable effect of the *Ananya* program on RMNCHN behaviours.

Interpretation of evaluation results must consider the large scale and ambitious scope of *Ananya* coupled with the short, two-year midline in the context of a planned five year initative. The *Ananya* interventions sought to saturate the health system at community and, to a lesser extent, at facility levels and through outreach by rolling out a number of interventions through various delivery platforms [13]. Timing of intervention introduction and ramp-up of implementation varied, however, and thus, many interventions were operational for even shorter periods of time. More time for continuation of program implementation under the *Ananya* management structure with intensive support to GoB implementation may have yielded more consistent and higher magnitude effects. Further, the midline evaluation was collected during the period when GoB implementation was transitioning to scale-up and thus intensity of implementation and *Ananya* program support to the GoB may have already been declining [13]. Additionally, improvements across multiple health indicators may have been limited by supply-side constraints. Last-mile supply chain and logistics management were challenging as many commodities were centrally procured, and supply chain management improvements for commodities like IFA tablets and modern contraception methods such as condoms and pills were not addressed in the first two years of the program. Similarly, improvements in complementary feeding behaviours through FLW counseling/advice may have been constrained by lack of household access to certain foods (eg, vegetables and meats). *Ananya* did not focus on complementary efforts such as providing nutritional supplements or promoting increases in household purchasing power that – when coupled with FLW counseling on complementary feeding and nutrition – might have generated more positive impact in this domain.

The evaluation design was not capable of identifying specific demand-side barriers to adoption. For example, babies being breastfed within one hour of birth for home deliveries decreased by four ppt, but we do not know if the constraint to adoption pertained to knowledge, attitude, skills, efficacy, or social norms. We conducted a realist evaluation in two districts of Bihar outside the *Ananya* pilot study area to characterise motivational mechanisms among ASHAs in Bihar [51]. Findings suggest that further efforts to nurture and sustain FLWs’ intrinsic motivation may be necessary for improving their performance in engaging beneficiaries in behaviour change to improve health. While the program sought to use data to help improve program approaches and GoB implementation and health impact, additional qualitative research, including assessment of implementation processes, and further emphasis on mixed methods research to gain insights into implementation successes and failures may have been instructive.

Other than two randomised controlled trials implemented during this period [19,20], it is not possible to attribute results to specific interventions or platforms. The Mathematica surveys were particularly limited in insight into the impact of facility-based interventions during this time period, with the exception of a small number of neonatal health behaviours for babies delivered in facilities (**Table 3**, **Table 4**). Facility-based interventions from 2012-2014 focused on filling gaps at facilities identified through self-assessment processes led by quality improvement teams [13,39,40], but quality improvement interventions were intensified and scaled up only after the study period covered by the Mathematica evaluation.

Collaborative efforts such as the PHC Performance Initiative [52] and *The Lancet* Global Health Commission on High-Quality Health Systems in the SDG Era [5] seek to catalyse improvements in the performance of health systems through identifying research gaps, informing the design of better measurement systems, and identifying and disseminating effective practices. These efforts underscore the need to address research gaps in health systems research, including evaluation of large-scale efforts to improve the quality of health services and measure the effects of quality-focused intervention designs on user experience, equity, and their impact on the performance of different components of the health system [5]. Similarly, research is needed to understand how performance measurement and management systems work in PHC systems [53], and the impact of socio-political dynamics on the adoption of health innovations and health-seeking behaviour [54]. Cost-effectiveness measures of large-scale RMNCHN programs are also needed.

To complement more traditional public health evaluations, program designers could utilise evaluation designs such as realist evaluations, which seek to discern “what works, for whom, in what respects, to what extent, and how” [55]. Given the challenge of evaluating programs such as *Ananya* which are comprised of multiple interventions and require great coordination among stakeholders, additional system-level approaches, tools, and research methods might also be applied, which recognise PHC systems as complex adaptive systems in which technical solutions alone are not adequate to induce the adoption of innovations [56]. Popular frameworks such as the World Health Organisation building blocks do not sufficiently capture key underlying and determinative processes underpinning the system’s components and actors. Beyond the concrete, formal, tangible, and well-understood “hardware” or component building blocks of health systems (eg,service delivery, workforce, financing), is the “software” of health systems, including ideas, interests, values and norms, power dynamics, and a host of other social and political factors including cultural determinants, informal rules, and communication patterns which play a key role in shaping health systems [57]. Research methods are required that can more effectively identify and analyse the multiple constraints facing FLWs in disseminating health information and inducing adoption of key health behaviours in communities, including the role of power structures, monetary and non-financial incentives, decision-making norms and traditions, the role of traditional health practices (eg, tradition of home delivery), and restrictive cultural and gender norms [49,58].

## CONCLUSION

The Mathematica evaluation offers an important contribution in its use of DID estimators to control for secular changes that impact health, and its broad assessment across the continuum of care and delivery platforms, in order to reliably analyse the contribution of *Ananya* during its pilot implementation period. Encouraging signs were seen in select RMNCHN indicators, but ultimately the duration and intensity of implementation were insufficient to achieve large-scale, statistically significant improvements in most health-related indicators. Of note, the evaluation provided early evidence of encouraging improvements in family planning uptake from very low baseline levels, due to program inputs. The finding of a significant increase in contraception among mothers of female children but not among mothers of males requires further investigation, and underscores the importance of bringing a gender lens to RMNCHN programming inputs and evaluations [59]. Impact of the program on health equity is reported separately [60]. Evaluations from complex, large-scale PHC programs such as *Ananya* offer the opportunity to advance the research agenda for PHC performance improvement at scale, and provide practical guidance to policymakers and practitioners seeking to invest in improving the performance of PHC systems in LMICs. This evaluation well illustrates the importance of taking secular changes into account and reinforces the need for theory-driven, gender- and equity-sensitive, policy-relevant, mixed-methods evaluations of large-scale health programs that inform action to improve primary health care system performance.

## Additional material

Online Supplementary Document
