# Supplementary material for: Impact of the Ananya program on reproductive, maternal, newborn and child health and nutrition in Bihar, India: early results from a quasi-experimental study
Source: J Glob Health. 2020 Dec 19;10(2):021002. doi: 10.7189/jogh.10.021002 (PMC7757842; doi:10.7189/jogh.10.021002)
Supplement: Online Supplementary Document [file jogh-10-021002-s001.pdf]

Supplementary Figure 1. Forest plot for difference-in-difference estimators for reproductive, maternal, newborn and child health and nutrition indicators by implementation platform for *Ananya* programme focus (intervention) and comparison districts at baseline (2012) and at midline (2014), Bihar, India, Mathematica data from maternal respondents with infants 0-11 months old.

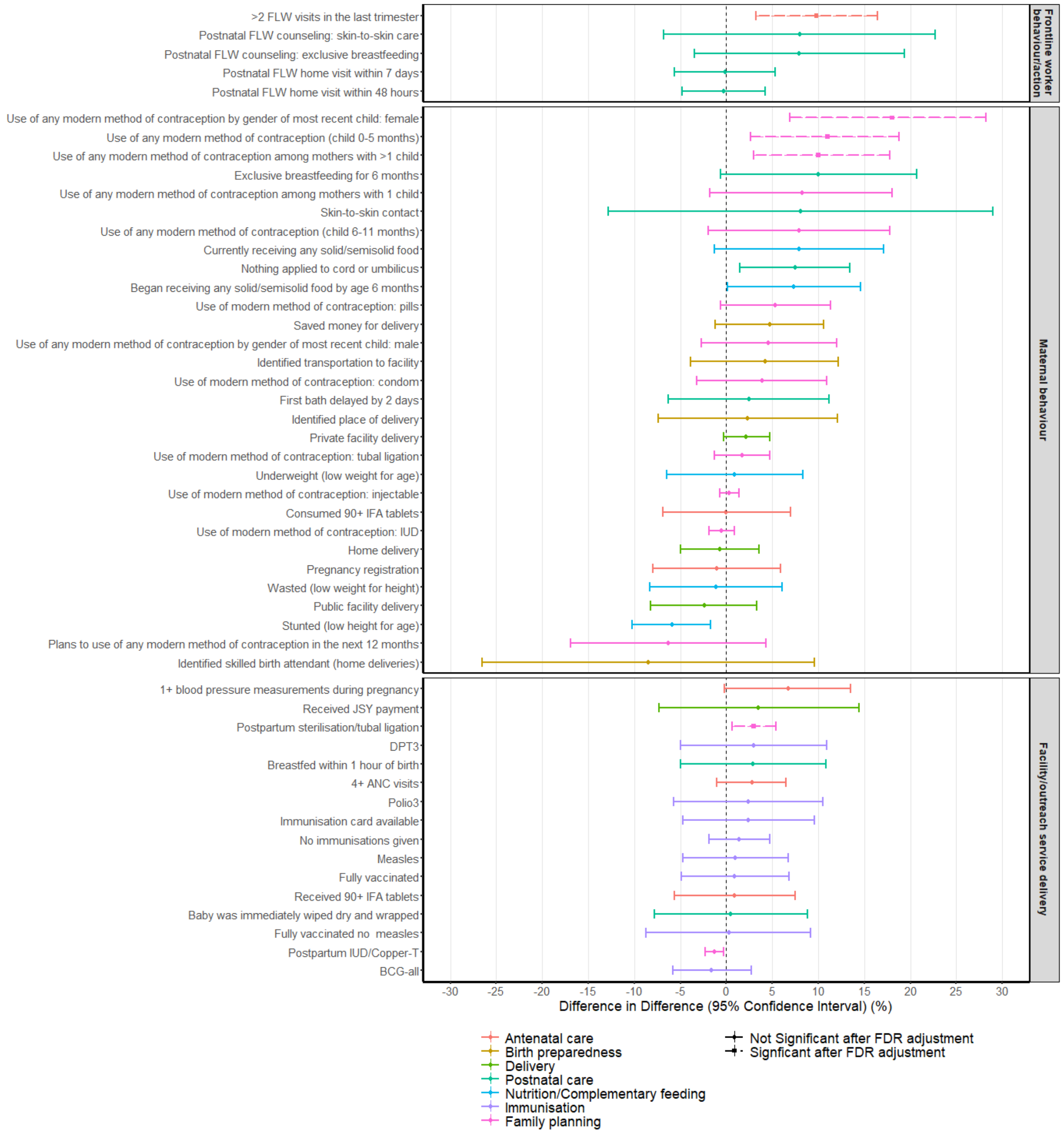

ANC, antenatal care; BCG, Bacillus Calmette–Guérin; DPT, diphtheria-pertussis-tetanus; FLW, frontline worker; IFA, iron-folic acid; IUD, intrauterine device; JSY, *Janani Avam Bal Suraksha Yojana*

Supplementary Figure 2: Forest plot for difference-in-difference estimators for reproductive, maternal, newborn and child health and nutrition indicators by the continuum of care and implementation platform for *Ananya* programme focus (intervention) and comparison districts at baseline (2012) and at midline (2014), Bihar, India, Mathematica data from maternal respondents with infants 0-11 months old.

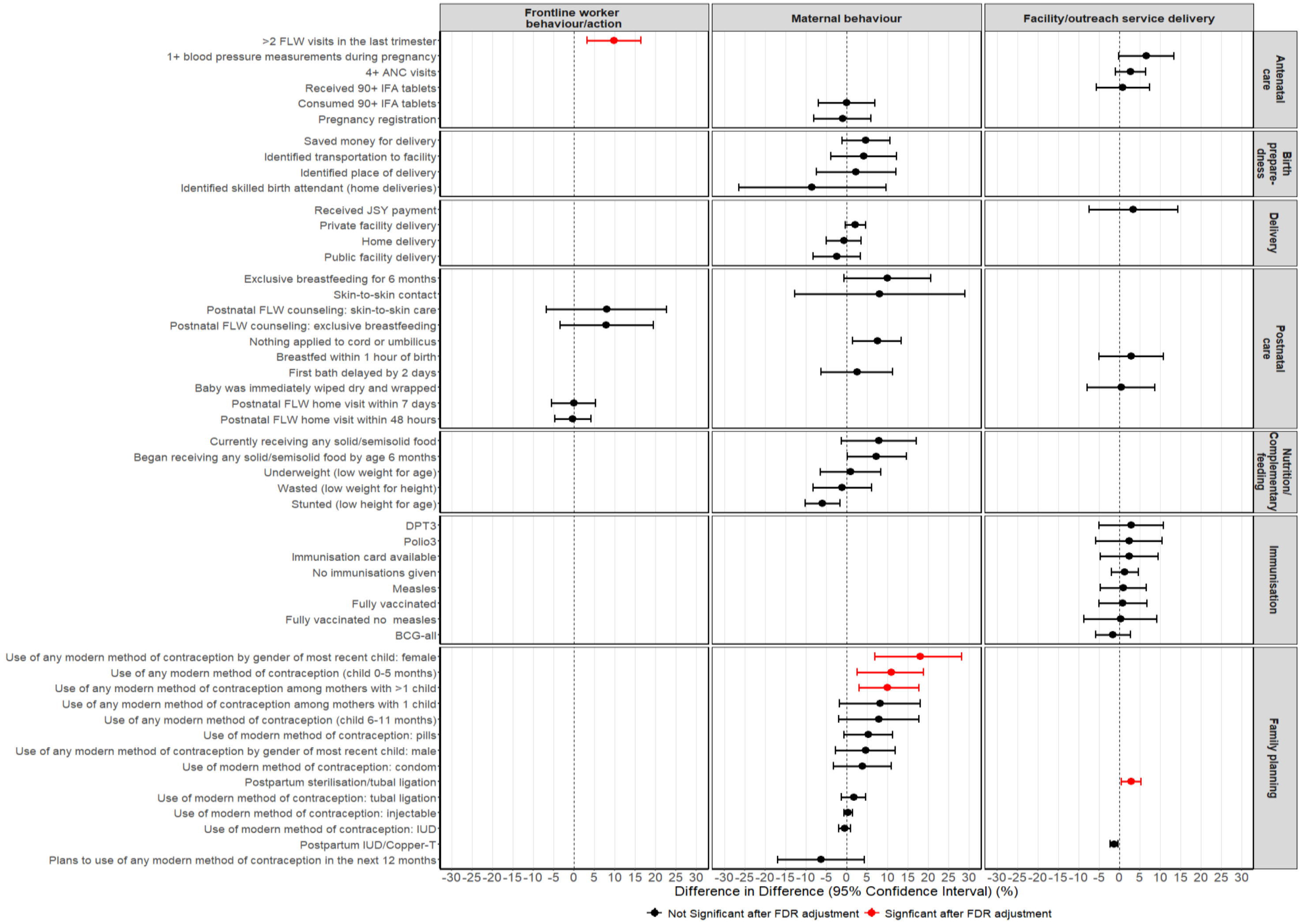

Supplementary Table 1. Indicators utilised in the Mathematica baseline (2012) and midline (2014) surveys of the *Ananya* programme in Bihar, India

<table>
<tr><th>Description</th><th>Platform</th><th>Continuum of Care</th><th>Platform</th><th>Time Period Available</th><th>Variable Name</th><th>Original Variables</th><th>Label of Original Variables</th><th>STATA Codes</th></tr>
<tr><td>Pregnancy registered</td><td>Maternal behavior</td><td>Antenatal care</td><td>Maternal behavior</td><td>Baseline+Midline</td><td>hh_pregreg</td><td>hh_pregreg</td><td colspan="2">pregnancy registered with anm, asha, or aww</td></tr>
<tr><td>Consumed 90 + IFA tablets</td><td>Facility/outreach service delivery</td><td>Antenatal care</td><td>Facility/outreach service delivery</td><td>Baseline+Midline</td><td>hh_ifatook_90more</td><td>hh_ifatook_90more</td><td>ifa tablets taken- at least 90</td><td></td></tr>
<tr><td>Received 90 + IFA tablets</td><td>Facility/outreach service delivery</td><td>Antenatal care</td><td>Facility/outreach service delivery</td><td>Baseline+Midline</td><td>hh_ifarcvd_90more</td><td>hh_ifarcvd_90more</td><td>ifa tablets received- at least 90</td><td></td></tr>
<tr><td>4 or more ANC checkups</td><td>Facility/outreach service delivery</td><td>Antenatal care</td><td>Facility/outreach service delivery</td><td>Baseline+Midline</td><td>hh_numvisit</td><td>t0_e06 t1_e06</td><td>How many times did you receive antenatal care during this pregnancy</td><td>egen hh_numvisit= rowmax(t0_e06 t1_e06); replace hh_numvisit=0 if hh_numvisit==.a| hh_numvisit==.x</td></tr>
<tr><td>At least 2 FLW visits in the last trimester</td><td>Frontline worker behavior/action</td><td>Antenatal care</td><td>Frontline worker behavior/action</td><td>Baseline+Midline</td><td>hh_flwvist_cat</td><td>hh_flwvist</td><td>times flw visited in last 3 months of pregnancy</td><td>gen hh_flwvist_cat=.
replace hh_flwvist_cat=1 if hh_flwvist>=2 & hh_flwvist !=.c & hh_flwvist !=.x
replace hh_flwvist_cat=0 if hh_flwvist<2 & hh_flwvist !=.
replace hh_flwvist_cat=. if hh_fcl_age_12plus==1
tab hh_flwvist_cat</td></tr>
</table>

								hh_flwvist
Identified skilled birth attendant	Maternal behavior	Birth preparedness	Maternal behavior	Baseline+Midline	hh_idsbam	t0_e29a t0_e33_a t1_e29a t1_e33a	identify and arrange for a skilled birth attendant, such as an anm, dai t;	egen hh_idsba = rowmax(t0_e29a t0_e33_a t1_e29a t1_e33a) gen hh_idsbam= 0 if hh_idsba==0 replace hh_idsbam= 1 if hh_idsba==1|hh_idsba==2 label var hh_idsbam "identified SBA spontaneous + probe"
Identified place of delivery	Maternal behavior	Birth preparedness	Maternal behavior	Baseline+Midline	hh_idfacm	t0_e33_g t1_e33f	identify health facility for the delivery?	egen hh_idfacm = rowmax(t0_e33_g t1_e33f)
Identified transportation to facility	Maternal behavior	Birth preparedness	Maternal behavior	Baseline+Midline	hh_idtransm	t0_e33_h t0_e32c t1_e33g t1_e32b	identify transportation to go to health facility?	egen hh_idtrans = rowmax(t0_e33_h t0_e32c t1_e33g t1_e32b) replace hh_idtransm= 1 if hh_idtrans==1|hh_idtrans==2 label var hh_idtransm "identified transportation for delivery"
Saved money for delivery	Maternal behavior	Birth preparedness	Maternal behavior	Baseline+Midline	hh_mondelm	t0_e32a t0_e33_f t1_e32a t1_e33e	save money for the delivery	egen hh_mondel = rowmax(t0_e32a t0_e33_f t1_e32a t1_e33e) gen hh_mondelm= 0 if hh_mondel==0 replace

								hh_mondelm= 1 if hh_mondel==1|hh_mondel==2 label var hh_mondelm "saved money for delivery"
Blood pressure measured at least once during pregnancy	Facility/outreach service delivery	Birth preparedness	Facility/outreach service delivery	Baseline+Midline	hh_ancproc_bp	hh_ancproc_bp	blood pressure taken at least once	
Home delivery	Maternal behavior	Delivery	Maternal behavior	Baseline+Midline	place_delivery_home	t0_f08 t1_f08	where did you deliver	egen hh_deliveryplace = rowmax(t0_f08 t1_f08)
Public facility delivery	Maternal behavior	Delivery	Maternal behavior	Baseline+Midline	place_delivery_public	t0_f08 t1_f08	where did you deliver	egen hh_deliveryplace = rowmax(t0_f08 t1_f08);gen place_delivery_public=1 replace place_delivery_public=0 if place_delivery_home==1|place_delivery_pvtngohospothsec==1
Private facility delivery: Private hospital/NGO hospital or clinic/ other private health facilities delivery	Maternal behavior	Delivery	Maternal behavior	Baseline+Midline	place_delivery_pvtngohospothsec	t0_f08 t1_f08	where did you deliver	egen hh_deliveryplace = rowmax(t0_f08 t1_f08)
Baby was immediately wiped dry and wrapped	Facility/outreach service delivery	Postnatal care	Facility/outreach service delivery	Baseline+Midline	hh_wipedryN	hh_wipedry	baby wiped dry and wrapped in cloth	gen hh_wipedryN = .c replace hh_wipedryN = 1 if hh_wipeddry

								== 1<br>replace hh_wipedryN = 0 if hh_wipeddry != 1<br>replace hh_wipedryN = . if hh_fcl_age_12plus==1
Postnatal visit within 48 hours by any FLW	Frontline worker behavior/action	Postnatal care	Frontline worker behavior/action	Baseline+Midline	hh_flw48	t0_g03a t0_g03 t1_g03_a t1_g03_a t1_g03_b	who came to visit you and the baby? was it the asha, aww, anm?: asha; how many days/weeks after the delivery did the first home visit take place;	gen hh_flw48=.<br>replace hh_flw48 = 1 if (t0_g03a==1 & t0_g03b<= 2) |(t1_g03_a== 0)|(t1_g03_a== 1& t1_g03_b<= 2)<br>replace hh_flw48 = 0 if hh_flw48 != 1<br>replace hh_flw48 = . if hh_fcl_age_12plus==1
Postnatal visit within 7 days by any FLW	Frontline worker behavior/action	Postnatal care	Frontline worker behavior/action	Baseline+Midline	hh_FLW7	t0_g04 t1_g04	how many times did an asha, aww, anm visit you at home during the first week	egen hh_FLW7=rowmax(t0_g04 t1_g04)<br>replace hh_FLW7=1 if hh_FLW7>=1<br>replace hh_FLW7=0 if hh_FLWPost==0
1st bath delayed by 2 days	Maternal behavior	Postnatal care	Maternal behavior	Baseline+Midline	hh_bath_2days	hh_bath_2days	thermal care: delay first bath at least two days	
Breastfed within 1 hour of birth	Facility/outreach service delivery	Postnatal care	Facility/outreach service delivery	Baseline+Midline	hh_bfed_1less	hh_bfed_1less	how long after birth was first feeding- less than 1 hour	
Received JSBY	Facility/outreach	Postnatal care	Facility/outreach	Baseline+Midline	hh_receive_js	hh_receive_jsy	received jsy	

payment	service delivery		service delivery		y		payment	
Nothing applied to cord or umbilicus	Maternal behavior	Postnatal care	Maternal behavior	Baseline+Midline	hh_nothingcord_umb	hh_nothingcord_umb	applied nothing to cord and umbilicus	
Postnatal FLW counseling (exclusive breastfeeding)	Frontline worker behavior/action	Postnatal care	Frontline worker behavior/action	Baseline+Midline	hh_advchdflw_all_brst	hh_advchdflw_all_brst	advice received from flw on child care- all, breast feed	
Postnatal FLW counseling (skin to skin care)	Frontline worker behavior/action	Postnatal care	Frontline worker behavior/action	Baseline+Midline	hh_advchdflw_all_skntskin	hh_advchdflw_all_skntskin	advice received from flw on child care- all, skin to skin care	
Skin to Skin contact	Maternal behavior	Postnatal care	Maternal behavior	Baseline+Midline	hh_skintoskin	hh_skintoskin	placed child in skin-to-skin contact	
Exclusive breast feeding for 6 months excluding water (self-reported)*	Maternal behavior	Postnatal care	Maternal behavior	Baseline+Midline	hh_bfedexcl_6to12_water	hh_bfedexcl_6to12_water	child exclusively breastfed through 6months excl water- 6 to 12 mo	
Stunted (low height for age, for infants 0-11 months)	Maternal behavior	Nutrition/Complementary feeding	Maternal behavior	Baseline+Midline	h_heightforage_z_cat	h_heightforage_z	height for age z-score	gen hh_heightforage_z_cat=. replace hh_heightforage_z_cat=1 if hh_heightforage_z<=-2 replace hh_heightforage_z_cat=0 if hh_heightforage_z>-2 replace hh_heightforage_z_cat=. if hh_fcl_age_12plus==1 replace hh_heightforage_z_cat=. if

								hh_heightforage_z==.a|hh_heightforage_z==.c|hh_heightforage_z==.d|hh_heightforage_z==.x
Wasted (low weight for height, for infants 0-11 months)	Maternal behavior	Nutrition/Complementary feeding	Maternal behavior	Baseline+Midline	hh_weightforage_z_cat	hh_weightforage_z	weight for age z-score	gen hh_weightforage_z_cat=. replace hh_weightforage_z_cat=1 if hh_weightforage_z<=-2 replace hh_weightforage_z_cat=0 if hh_weightforage_z>-2 replace hh_weightforage_z_cat=. if hh_fcl_age_12plus==1 replace hh_weightforage_z_cat=. if hh_weightforage_z==.a|hh_weightforage_z==.c|hh_weightforage_z==.d|hh_weightforage_z==.x
Underweight (low weight for age, for infants 0-11 months)	Maternal behavior	Nutrition/Complementary feeding	Maternal behavior	Baseline+Midline	hh_weightforheight_z_cat	hh_weightforheight_z	weight for height z-score	gen hh_weightforheight_z_cat=. replace hh_weightforheight_z_cat=1 if hh_weightforheig

<table>
<tr><td></td><td></td><td></td><td></td><td></td><td></td><td></td><td></td><td>ht_z<=-2
replace hh_weightforheight_z_cat=0 if hh_weightforheight_z>-2
replace hh_weightforheight_z_cat=. if hh_fcl_age_12plus==1
replace hh_weightforheight_z_cat=. if hh_weightforheight_z==.a|hh_weightforheight_z==.c|hh_weightforheight_z==.d|hh_weightforheight_z==.x</td></tr>
<tr><td>Began receiving any solid/semisolid food by age 6 months*</td><td>Maternal behavior</td><td>Nutrition/Complementary feeding</td><td>Maternal behavior</td><td>Baseline+Midline</td><td>hh_babyfdcf_6more</td><td>hh_babyfdcf_6more</td><td colspan="2">any babyfood-based complementary feeding yesterday - child 6 months or older</td></tr>
<tr><td>Currently receiving any solid/semisolid food*</td><td>Maternal behavior</td><td>Nutrition/Complementary feeding</td><td>Maternal behavior</td><td>Baseline+Midline</td><td>hh_anycf_6more</td><td>hh_anycf_6more</td><td colspan="2">any complementary feeding-child 6 months/more (>6 months)</td></tr>
<tr><td>BCG-all</td><td>Facility/outreach service delivery</td><td>Immunization</td><td>Facility/outreach service delivery</td><td>Baseline+Midline</td><td>hh_vac_bcg</td><td>hh_vac_bcg</td><td>received bcg</td><td></td></tr>
<tr><td>No immunizations given</td><td>Facility/outreach service delivery</td><td>Immunization</td><td>Facility/outreach service delivery</td><td>Baseline+Midline</td><td>hh_noimmun</td><td>hh_noimmun</td><td>no immunization received</td><td></td></tr>
<tr><td>Immunization card available</td><td>Facility/outreach service delivery</td><td>Immunization</td><td>Facility/outreach service delivery</td><td>Baseline+Midline</td><td>hh_card_avail</td><td>hh_card_avail</td><td>Immunization card available</td><td></td></tr>
<tr><td>Fully vaccinated</td><td>Facility/outreach service delivery</td><td>Immunization</td><td>Facility/outreach service delivery</td><td>Baseline+Midline</td><td>hh_vac_full</td><td>hh_vac_full</td><td>Fully vaccinated</td><td></td></tr>
<tr><td>Measles</td><td>Facility/outreach service delivery</td><td>Immunization</td><td>Facility/outreach service delivery</td><td>Baseline+Midline</td><td>hh_vac_msls</td><td>hh_vac_msls</td><td>received measles</td><td></td></tr>
</table>

Fully vaccinated no measles	Facility/outreach service delivery	Immunization	Facility/outreach service delivery	Baseline+Midline	hh_vac_flnomsls	hh_vac_flnomsls	Fully vaccinated no measles	
Polio3	Facility/outreach service delivery	Immunization	Facility/outreach service delivery	Baseline+Midline	hh_vac_polio3	hh_vac_polio3	received polio 3	
DPT3	Facility/outreach service delivery	Immunization	Facility/outreach service delivery	Baseline+Midline	hh_vac_dpt3	hh_vac_dpt3	received dpt3	
Plans to use of any modern method of contraception in the next 12 months for currently non-users	Maternal behavior	Family planning	Maternal behavior	Baseline+Midline	hh_intendfp_nonuser	hh_intendfp_nonuser	intend to use contraceptives in next 12 months (non-users)	
Postpartum IUD/Copper-T	Facility/outreach service delivery	Family planning	Facility/outreach service delivery	Baseline+Midline	hh_ppart_iud	hh_ppart_iud	facility delivery: postpartum iud	
Use of IUD	Maternal behavior	Family planning	Maternal behavior	Baseline+Midline	hh_modmthdtyp_iud_all	hh_modmthdtyp_iud_all	modern method used - iud all	
Use of Injectables	Maternal behavior	Family planning	Maternal behavior	Baseline+Midline	hh_modmthdtyp_injct_all	hh_modmthdtyp_injct_all	modern method used - injectables all	
Use of Tubal ligation	Maternal behavior	Family planning	Maternal behavior	Baseline+Midline	hh_modmthdtyp_fs_all	hh_modmthdtyp_fs_all	modern method used - female sterilization:all	
Postpartum sterilization/Tubal ligation	Facility/outreach service delivery	Family planning	Facility/outreach service delivery	Baseline+Midline	hh_ppart_tl	hh_ppart_tl	facility delivery: postpartum sterilization	
Use of Condom	Maternal behavior	Family planning	Maternal behavior	Baseline+Midline	hh_modmthdtyp_cndm_all	hh_modmthdtyp_cndm_all	modern method used - condoms all	
Modern Contraception (Male child)	Maternal behavior	Family planning	Maternal behavior	Baseline+Midline	hh_modmthd_all; boys	hh_modmthd_all	gen boys=.c<br>replace boys=1 if d_gender_fcl_chld==1<br>replace boys=0 if d_gender_fcl_chld==2<br><br>gen girls=.c<br>replace girls=1 if d_gender_fcl_chld==2<br>replace girls=0 if d_gender_fcl_chld==1	

Use of Pills	Maternal behavior	Family planning	Maternal behavior	Baseline+Midline	hh_modmthdtyp_pill_all	hh_modmthdtyp_pill_all	modern method used - pills all
Modern Contraception (6-11 months)	Maternal behavior	Family planning	Maternal behavior	Baseline+Midline	hh_modmthd_6to11	hh_modmthd_6to11	currently use a modern method; gave birth 6-11 months
Modern Contraception (Mothers with 1 child)	Maternal behavior	Family planning	Maternal behavior	Baseline+Midline	hh_numkids1only	hh_numkids1only	gen hh_numkids1only=.c<br>replace hh_numkids1only = 1 if hh_numkids == 1<br>replace hh_numkids1only = 0 if hh_numkids >= 2
Modern Contraception (Mothers with more than 1 child)	Maternal behavior	Family planning	Maternal behavior	Baseline+Midline	hh_numkids1andmo	hh_numkids1andmo	gen hh_numkids1andmo=.c<br>replace hh_numkids1andmo = 0 if hh_numkids == 1<br>replace hh_numkids1andmo = 1 if hh_numkids >= 2
Modern Contraception (0-5 months)	Maternal behavior	Family planning	Maternal behavior	Baseline+Midline	hh_modmthd_6less	hh_modmthd_6less	currently use a modern method; gave birth 0-5 months
Modern Contraception (Female child)	Maternal behavior	Family planning	Maternal behavior	Baseline+Midline	hh_modmthd_all; girls	hh_modmthd_all	gen girls=.c<br>replace girls=1 if d_gender_fcl_chld==2<br>replace girls=0 if d_gender_fcl_chld==1<br><br>gen girls=.c<br>replace girls=1 if d_gender_fcl_chld==2<br>replace girls=0 if d_gender_fcl_chld==1

ANC, antenatal care; BCG, Bacillus Calmette–Guérin; DPT, diphtheria-pertussis-tetanus; FLW, frontline worker; IFA, iron-folic acid; IUD, intrauterine device; JSY, *Janani Avam Bal Suraksha Yojana*; NGO, non-governmental organisation

Supplementary Table 2. Indicators for the *Ananya* programme categorised by implementation platform and continuum of care for reproductive, maternal, newborn and child health and nutrition, Bihar, India, Mathematica data from maternal respondents with infants 0-11 months old.

Implementation platform (columns) vs Continuum of care (rows)	Frontline worker (FLW) behavior/action	Maternal behaviour	Facility/outreach service delivery
**Antenatal care**	• >2 FLW visits in the last trimester	• Consumed 90+ iron-folic acid (IFA) tablets<br>• Pregnancy registration	• 4+ antenatal care visits<br>• 1+ blood pressure measurements during pregnancy<br>• Received 90+ IFA tablets
**Birth preparedness**		• Saved money for delivery<br>• Identified place of delivery<br>• Identified transportation to facility<br>• Identified skilled birth attendant (home deliveries)	
**Delivery (childbirth)**		• Home delivery<br>• Delivery in public facility<br>• Delivery in private facility	• Received *Janani Avam Bal Suraksha Yojana* (JBSY) payment
**Postnatal care**	• Postnatal FLW home visit within 48 hours<br>• Postnatal FLW home visit within 7 days<br>• Postnatal FLW counseling: skin-to-skin care<br>• Postnatal FLW counseling: exclusive breastfeeding	• Exclusive breastfeeding for 6 months[a]<br>• Nothing applied to cord or umbilicus<br>• Skin-to-skin contact<br>• First bath delayed by 2 days	• Breastfed within 1 hour of birth<br>• Baby was immediately wiped dry and wrapped
**Child nutrition/ Complementary feeding**		• Currently receiving any solid/semisolid food<br>• Began receiving any solid/semisolid food by age 6 months<br>• Stunted (low height for age)<br>• Wasted (low weight for height)<br>• Underweight (low weight for age)	
**Child immunisation**			• Immunisation card available<br>• No immunisations given<br>• Bacillus Calmette–

			Guérin (BCG)<br>• Diphtheria-pertussis-tetanus (DPT)3<br>• Measles<br>• Polio3<br>• Fully vaccinated no measles<br>• Fully vaccinated
**Family planning**		• Plans to use of any modern method of contraception in the next 12 months<br>• Use of any modern method of contraception (child 0-5 months)<br>• Use of any modern method of contraception (child 6-11 months)<br>• Use of modern method of contraception: condom<br>• Use of modern method of contraception: IUD<br>• Use of modern method of contraception: pills<br>• Use of modern method of contraception: tubal ligation<br>• Use of modern method of contraception: injectable<br>• Use of any modern method of contraception among mothers with 1 child<br>• Use of any modern method of contraception among mothers with >1 child<br>• Use of any modern method of contraception by gender of most recent child: male<br>• Use of any modern method of contraception by gender of most recent child: female	• Postpartum sterilisation/tubal ligation<br>• Postpartum intrauterine device (IUD)/Copper-T

[*]Excluding water (self-report for child 6-11 months)
[a]Percentage unless otherwise specified
[b]Median used due to non-normal data
[c]SES quartile determined using National Family Health Survey-3 methods

Supplementary Table 3. Sample size and focal child's age group for *Ananya* programme indicators in Table 3, Bihar India, Mathematica data, 2012 and 2104.

		**Baseline 2012**		**Midline 2014**	
	**Focal Child's Age Group**	**Comparison (N)**	**Intervention (N)**	**Comparison (N)**	**Intervention (N)**
**Total Cohort**	0-11 month	9406	2978	8562	3092
	0-5 month	5641	1814	4967	1782
	6-11 month	3765	1164	3595	1309
**Antenatal Care**					
Antenatal Checkups					
4 or more antenatal care checkups	0-11 month	9406	2978	8562	3092
BP measured at least once during pregnancy	0-11 month	9406	2978	8562	3092
More than 2 frontline worker (FLW) home visits in the last trimester	0-11 month	9343	2967	8562	3089
Iron-Folic Acid					
Received 90 + tablets	0-11 month	9406	2978	8562	3026
Consumed 90 + tablets	0-11 month	9405	2978	8562	3025

**Birth Preparedness**					
Identified place of delivery	0-11 month	6687	2107	2531	777
Identified skilled birth attendant	0-11 month	4193	1286	2531	777
Saved money for delivery	0-11 month	6698	2107	2933	2668
Identified transportation to facility	0-11 month	6698	2107	2933	2668
Pregnancy registered	0-11 month	9406	2973	8562	3092
**Delivery (Childbirth)**					
Place of Delivery	0-11 month	9406	2978	8562	3092
Home delivery	0-11 month	9406	2978	8562	3092
Facility delivery	0-11 month	9406	2978	8562	3092
Public	0-11 month	9406	2978	8562	3092
Private	0-11 month	9406	2978	8562	3092
Received *Janani Avam Bal Suraksha Yojana* (JBSY) payment	0-11 month	5794	1944	6136	2379
**Postnatal Care**					
Postnatal FLW Visit					
Postnatal visit within 48 hours by any FLW	0-11 month	9406	2978	8562	3092
Postnatal visit within 7 days by any FLW	0-11 month	9406	2978	8562	3092
Postnatal FLW counseling					
Skin-to-skin (kangaroo) care	0-11 month	6903	2146	8562	3092
Exclusive breastfeeding	0-11 month	6903	2146	8562	3092
Neonatal health-related behaviors					
Baby was immediately wiped dry and wrapped	0-11 month	9406	2978	8562	3092
Nothing applied to cord or umbilicus	0-11 month	9406	2891	8262	2794
Skin to skin contact [a]	0-11 month	9406	2954	8527	3085
1st bath delayed by 2 days	0-11 month	9404	2950	8506	3063
Breastfeeding					
Breastfed within 1 hour of birth	0-11 month	9364	2961	8512	3063
Exclusive breast feeding for 6 months excluding water (self-report for child 6-11 month)	6-11 month	3765	1160	3592	1301
**Child Nutrition**					
Complementary feeding among children aged 6-11 months					
Currently receiving any solid/semisolid food	6-11 month	3759	1164	3595	1309
Began receiving any solid/semisolid food by age 6 months	6-11 month	3759	1164	3595	1309
Indicators for undernutrition in children					
Stunted (low height for age)	0-11 month	3123	954	7571	2617
Wasted (low weight for height)	0-11 month	3245	1004	7643	2625

Underweight (low weight for age)	0-11 month	3122	974	7342	25
**Child Immunisation**					
Immunisation card available	0-11 month	9406	2978	8562	3092
No immunisations given	0-11 month	9406	2978	8562	3092
Individual immunisations					
Bacillus Calmette–Guérin (BCG)	0-11 month	9286	2948	8520	3082
Diphtheria-pertussis-tetanus (DPT)3	0-11 month	9223	2946	8520	3082
Measles	0-11 month	9055	2858	8520	3082
Polio3	0-11 month	8736	2769	8520	3082
Fully vaccinated no measles	0-11 month	8733	2769	8520	3082
Fully vaccinated	0-11 month	8544	2681	8520	3082
**Family Planning**					
Procedures performed after delivery					
Postpartum sterilisation/ Tubal lligation	0-11 month	5794	1945	6136	2379
Postpartum intrauterine device (IUD)/Copper-T	0-11 month	5794	1945	6136	2379
Plans to use of any modern method of contraception in the next 12 months	0-11 month	6705	2328	7251	2455
Use of any modern method of contraception by age of child	0-11 month	9331	2952	8474	3047
0-5 months	0-5 month	5629	1811	4957	1778
6-11 months	6-11 month	3702	1141	3517	1269
Use of any modern method of contraception by method					
Condom	0-11 month	9331	2952	8474	3047
IUD	0-11 month	9331	2952	8474	3047
Pills	0-11 month	9331	2952	8474	3047
Tubal ligation	0-11 month	9331	2952	8474	3047
Injectables	0-11 month	9331	2952	8474	3047
Use of any modern method of contraception by parity					
Mothers with 1 child	0-11 month	6347	2054	5818	2142
Mothers with more than 1 child	0-11 month	2984	898	2656	905
Use of any modern method of contraception by gender of most recent child					
Male	0-11 month	4864	1546	4543	1667
Female	0-11 month	4467	1406	3931	1380

districts and 267 women in intervention districts.

Supplementary Table 4. Health status indicators in India and Bihar based on Sample Registration System (SRS) national survey data.

**Indicator**	**India**	**Bihar**			
	**Most recent estimate**	**Trend (year and source)**			
Maternal mortality ratio (per 100,000 live births)	167 (2011-2013)	312 (2004-2006)	261 (2007-2009)	219 (2010-2012)	208 (2011-2013)
Infant mortality rate (0-1 year) (per 1000 live births)	39 (2014)	48 (2010)	44 (2011)	43 (2012)	42 (2014)
Neonatal mortality rate (0-28 days) (Per 1000 live births)	26 (2014)	31 (2010)	29 (2011)	28 (2012)	27 (2014)
Early neonatal mortality rate (within 7 days of birth) (per 1000 live births)	20 (2014)	27 (2010)	25 (2011)	23 (2012)	22 (2014)
Under-5 mortality rate (per 1000 live births)	45 (2014)	64 (2010)	59 (2011)	57 (2012)	53 (2014)
Total fertility rate	2.3 (2014)	3.7 (2010)	3.6 (2011-2012)	3.5 (2012-2013)	3.2 (2014)

Supplementary Table 5. Trends in reproductive, maternal, newborn and child health and nutrition indicators in Bihar, India, based on Annual Health Survey (AHS) data.

Indicator	AHS 1-2011	AHS 2-2012	AHS 3-2013
Mothers who had at least 3 antenatal care visits (%)	34	34	37
Mothers who received postnatal care within 2 days of delivery (%)	61	60	61
Institutional births (%)	48	52	55
Percent of women who received 2 or more tetanus toxoid injections	84	85	85
Mothers who consumed iron-folic acid for 100+ days during pregnancy (%)	10	10	13
Children under age 3 years breastfed within our hour of birth (%)	30	35	37
Children under age 6 months exclusively breastfed (%)	29	30	31
Children age 6-8 months receiving solid or semi-solid food and breast milk (%)	6	7	7
Children 12-23 months fully immunisized [Bacillus Calmette–Guérin (BCG), 3 injections of diphtheria-pertussis-tetanus (DPT), 3 doses of polio excluding polio 0 and measles) (%)	65	66	70
Modern contraceptive prevalence rate (%)	34	38	37
